# Evaluation of the Anti-Tumor Activity of Small Molecules Targeting Eph/Ephrins in APC ^min^/J Mice

**DOI:** 10.3390/ph13040069

**Published:** 2020-04-16

**Authors:** Miriam Corrado, Carmine Giorgio, Elisabetta Barocelli, Giuseppe Vittucci Marzetti, Anna Maria Cantoni, Rosanna Di Lecce, Matteo Incerti, Riccardo Castelli, Alessio Lodola, Massimiliano Tognolini

**Affiliations:** 1 Food and Drug Department, University of Parma, Viale delle Scienze 27/a, 43124 Parma, Italy; 2 Department of Sociology and Social Research, University of Milano-Bicocca, Via Bicocca degli Arcimboldi 8, 20126 Milan, Italy; 3 Department of Veterinary Science, University of Parma, Strada del Taglio 10, 43126 Parma, Italy

**Keywords:** Eph-ephrin system, EphA receptors, EphB receptors, Eph antagonist, UniPR129, APC ^min^/J mice, colorectal cancer (CRC), Familial adenomatous polyposis (FAP)

## Abstract

The Eph receptors are the largest receptors tyrosine kinases (RTKs) family in humans and together with ephrin ligands constitute a complex cellular communication system often dysregulated in many tumors. The role of the Eph-ephrin system in colorectal cancer (CRC) has been investigated and different expression of Eph receptors have been associated with tumor development and progression. In light of this evidence, we investigated if a pharmacological approach aimed at inhibiting Eph/ephrin interaction through small molecules could prevent tumor growth in APC ^min^/J mice. The 8-week treatment with the Eph-ephrin antagonist UniPR129 significantly reduced the number of adenomas in the ileum and decreased the diameter of adenomas in the same region. Overall our data suggested as UniPR129 could be able to slow down the tumor development in APC ^min^/J mice. These results further confirm literature data about Eph kinases as a new valuable target in the intestinal cancer and for the first time showed the feasibility of the Eph-ephrin inhibition as a useful pharmacological approach against the intestinal tumorigenesis. In conclusion this work paves the way for further studies with Eph-ephrin inhibitors in order to confirm the Eph antagonism as innovative pharmacological approach with preventive benefit in the intestinal tumor development.

## 1. Introduction

Colorectal cancer (CRC) is the third most deadly cancer in the world, affecting thousands of people every year, especially in developed countries due to unhealthy western diet and physical inactivity [1,2,3]. Early diagnosis, improvements in surgical interventions and the availability of new drugs allowed to reduce the incidence and increased the average survival time for advanced CRC. However further efforts are still necessary due to the high rate mortality of advanced CRC patients, which usually die within three years [1].

Several studies have investigated the role of the Eph-ephrin system in CRC for last decades, even if it has not been completely understood yet. Different expression profile of Eph (Erythropoietin-Producing Hepatocellular carcinoma) receptors have been associated with CRC tumor development, tumor progression and related to the poor prognosis of patients. 

The Eph receptors are the largest receptors tyrosine kinases (RTKs) family in humans and they are divided in two classes, A (EphA1–A8, EphA10) and B (EphB1–B4, EphB6), based on sequence homology and binding affinity for their ligands, ephrins, which are membrane-anchored proteins. Ephrins are also classified in As and Bs where the former have a glycosyl-phosphatidylinositol-linkage whereas the latter contain a transmembrane domain. The interaction between an Eph receptor and an ephrin ligand generates a bidirectional signaling between the Eph-bearing cells (the forward signaling) and the ephrin-bearing cell (the reverse signaling) [4], leading to the activation of different downstream signal transduction cascades [5]. Generally, this system plays a key role in the normal intestine, as it controls the crypt architecture by regulating the positioning of the cells along crypt-villus axis and the proliferation of intestinal stem cells. EphB receptors and ephrin-B ligands are expressed in an opposite gradient along the intestinal crypts, with high EphB2 and EphB3 expression at the bottom of the crypts in which intestinal stem cells lie, and high ephrin-B2 and ephrin-B1 expression in more differentiated cells at the top in the villi [6]. 

Many studies have observed an altered expression of Eph receptors in colorectal cancers, especially for EphA2, EphA1, EphB4, and EphB2. High EphA2 levels have been found in the early stages of disease and associated with poor overall survival of patients. The EphA2 overexpression together with increased microvessel formation have been documented in primary colorectal human cancer specimens, suggesting a pro-angiogenic role for this receptor, especially in the earlier stages [7]. Finally, EphA2 overexpression was also recently correlated with an increased migratory and invasive capabilities of cancer cells. In fact, the down-regulation of EphA2 by means of RNAi or by ephrin-A1-Fc was able to reduce, at least in vitro, the cellular migration and invasion [8].

Besides EphA2, particularly worth mentioning is the EphA1 expression in CRC, reason why it was proposed as a prognostic marker in CRC. This receptor appears upregulated during the early stages while it is downregulated in the late stages such as metastatic and grade III phases [9]. 

As regards EphB receptors, EphB4 and EphB2 showed an opposite expression in colon cancer cells. The EphB4 expression relates with stage and grade of colon cancer, contrary to EphB2, whose expression is reduced [10] with the tumor progression, and it is firstly abundant on colon progenitor cells [11]. The levels of these two receptors are regulated by the Wnt/β-catenin/T-cell factor 4 pathway through the usage of different coactivators, as p300 for EphB2 and CBP for EphB4 [10]. This switch in the expression of EphB4 and EphB2 would provide survival advantages to tumor cells during tumor progression. EphB4 would seem to protect from the innate tumor necrosis factor-induced apoptosis and to support the tumor growth. EphB4 would promote the tumor angiogenesis by interacting with ephrin-B2 expressed on tumor vasculature [10]. In light of these findings, it was thought to evaluate the effects of the Eph-ephrin antagonism in a model of intestinal tumorigenesis, by using C57BL6/J mice carrying the APC ^min^ mutation. This strain spontaneously develops a large number of adenomas and sporadic carcinoma especially in the small intestine and generally do not survive for more than 120 days [12,13]. These mice have been extensively used for the study of intestinal tumor pathogenesis and for the assessment of novel agents for cancer prevention and treatment [12,13,14].

As the suppression of EphA2 through gene knockdown in APC ^min^/J mice was accompanied to the development of fewer and smaller tumors in the small and large intestine [15], it was investigated if the same results could be achieved with compounds which block Ephs activation, through the inhibition of the interaction with the ephrin ligands. We used three competitive and reversible pan Eph antagonists (UniPR129, UniPR139, and UniPR1331) which are able to prevent the EphA2-ephrinA1 interaction with high potency. UniPR129 is the L-homo tryptophan conjugate of Lithocholic acid endowed with a submicromolar Ki (Ki = 390 nM) [16], and UniPR139 is a compound stemmed from UniPR129 structure, slightly less potent than the parent compound (Ki = 950 nM) but oral bioavailable in mice [17]. UniPR129 was previously observed to not be bioavailable after oral administration [16,18], because of extensive first pass hepatic metabolism [18]. In this study it was investigated if it could exert a local effect in the intestine. Finally, the third tested compound was UniPR1331 (Ki = 1430 nM), the L-homo tryptophan conjugate of cholenic acid, which has shown to reduce tumor growth in several models of glioblastoma [19,20].

## 2. Results

### 2.1. Toxicity

UniPR129, UniPR139 and UniPR1331 were tested in an animal model of intestinal tumorigenesis, based on the usage of APC ^min^/J mice, which spontaneously develop multiple and large adenomas over intestinal tract due to a gene mutation [12]. In our experiment, we studied the initial phase of the polyposis before the disease significantly affects the healthy state of the animals reducing body weight and inducing severe anemia [12]. Five weeks old mice have been treated with the three compounds at 30 mg/kg dose for 8 weeks every other day following previous observations [19,21]. The weight of the mice has been monitored from the beginning of the treatment until the end of the study to follow the course of the disease and to detect potentially toxic effects of compounds. 

**Figure 1:**
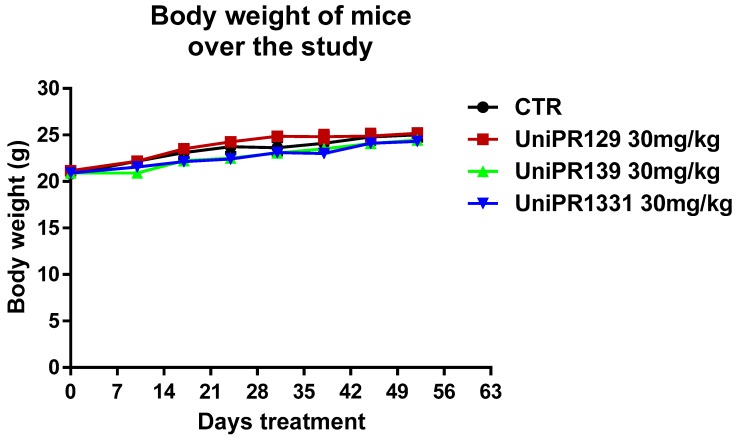
Trend of mice weight over the study. The mean weight of mice, recorded once a week in each experimental group (n = 7), was represented.

As shown in Figure 1, each mice group did not slim down throughout the experimentation. The body weight increase of the control group corroborated the early stage of the polyposis in our experimental settings. The safety of the compounds was also confirmed by toxicological studies carried out at the end of the study. In fact, the treatment with 30 mg/kg of UniPR129, UniPR139, and UniPR1331 was well tolerated from the animals and it did not lead to modifications of hematological parameters and blood chemistry. Only UniPR139 determined a meaningful increase of neutrophils percentage and a reduction of lymphocytes one (Table 1).

### 2.2. Effects of Eph-Ephrin Antagonists on the Number of Adenomas: “Statistical Considerations”

**Figure 2:**
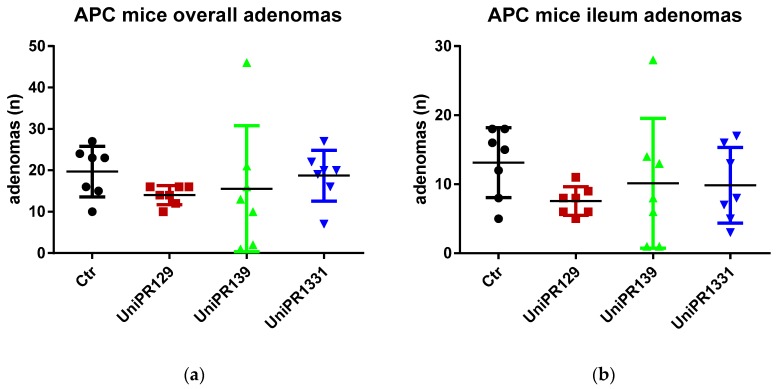
(**a**) Comparison of the total number of adenomas developed in the intestine between control and treatments groups; (**b**) Comparison of the number of adenomas developed in the ileum section between control and treatments groups. n = 7.

Table 2 and Figure 2 summarize the number of adenomas developed by APC ^min^/J mice in the whole intestine and in the ileum for the control group and the different treatments (UniPR129, UniPR1331, UniPR139). 

Although the standard one-way ANOVA does not reject the null hypothesis the mean being equal for all the groups (*p*-value = 0.5977 and 0.42 for the overall intestine and the ileum adenomas, respectively), the homoscedasticity assumption is clearly violated (the Bartlett’s tests for equal variances reject the null at the 5% significance level, with *p*-values equal to 0.001 and 0.015 for the overall intestine adenomas and the ileum adenomas, respectively). In fact, the *p*-values of the (simulated) W tests, a version of the ANOVA test robust to heteroscedasticity, are significantly lower although still slightly above 10% (0.133 and 0.138, respectively [22,23]). 

As far as the comparison between the control group and the group treated with UniPR129 is concerned, the evidence is much stronger. The ANOVA test is equivalent to a *F* test for the overall significance of a regression of the number of adenomas on a constant and three dummies (one for each treatment group). If we run such a regression for the ileum adenomas, the t test for the significance of the coefficient attached to the group treated with UniPR129 does not reject the null at the 10% (and it is the only coefficient which is statistically significant) and in cases like this the *t* test should be preferred as it has more power, i.e., there is higher probability to reject the null when the null is false.

The same results are achieved when we consider the pairwise comparisons between the control group and the treated groups (UniPR129, UniPR1331, UniPR139), carrying out two-sample t tests (assuming equal and unequal variance) to test the null of no difference in means. As it is apparent from Table 3, which reports the two-tailed *p*-values of the tests, the only treatment that exhibits statistically significant differences with respect to the control group (at the 5% level) is UniPR129.

Another possibility to make the test robust to unknown forms of heteroscedasticity is to run the regression of the number of adenomas on a constant and the dummies for the treated groups by using the Huber/White estimator of variance (which is a consistent estimator, although admittedly with no properties in finite samples and the sample size in our case is rather small) and carry out a Wald test for the overall significance of the regression. If we carry out such test, we reject the null that the coefficients attached to the dummies are zero (no effect of treatments) at the 10% significance level (*p*-value = 0.07 for both overall and ileum adenomas) and this result is driven only by the significance of the coefficient attached to the group treated with UniPR129, the only statistically significant (with *p*-values of *t* test equal to 0.029 and 0.012 for overall and ileum adenomas, respectively). 

### 2.3. Effect of UniPR129 on Adenomas

**Figure 3:**
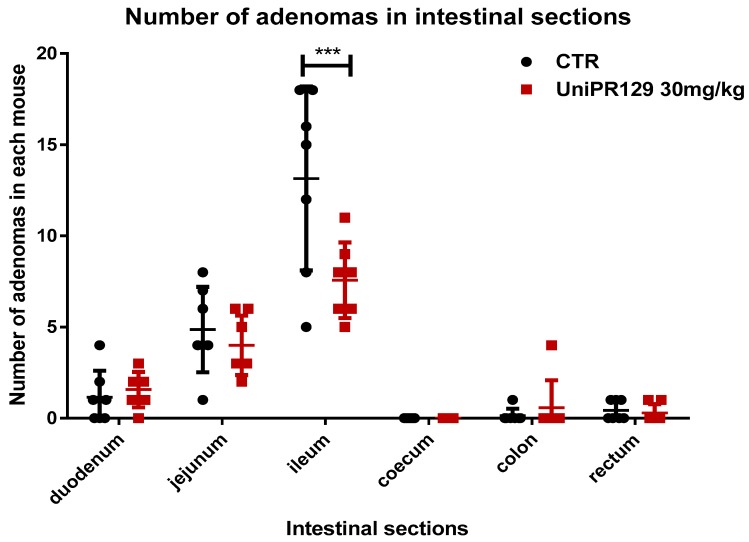
Comparison of the number of tumors developed in different sections of the intestine between control and UniPR129 group. Two-way ANOVA followed by Bonferroni’s post-test was used to compare control to UniPR129 group. *** *p* < 0.001, n = 7.

As statistical analysis revealed that only the oral administration of UniPR129 at the dosage of 30 mg/kg for 8 weeks significantly decreased the formation of adenomas, we decided to focus on this compound and to neglect the study of UniPR139 and UniPR1331. Thus, the number of adenomas in different section of the intestine was assessed. As shown in Figure 3, UniPR129 reduced the formation of adenomas in the ileum of 1.6 times. The mean number of adenomas shifted from 13.14 ± 5.05 (SD) for the control group to 7.57 ± 2.07 (SD) for the treatment group.

**Figure 4:**
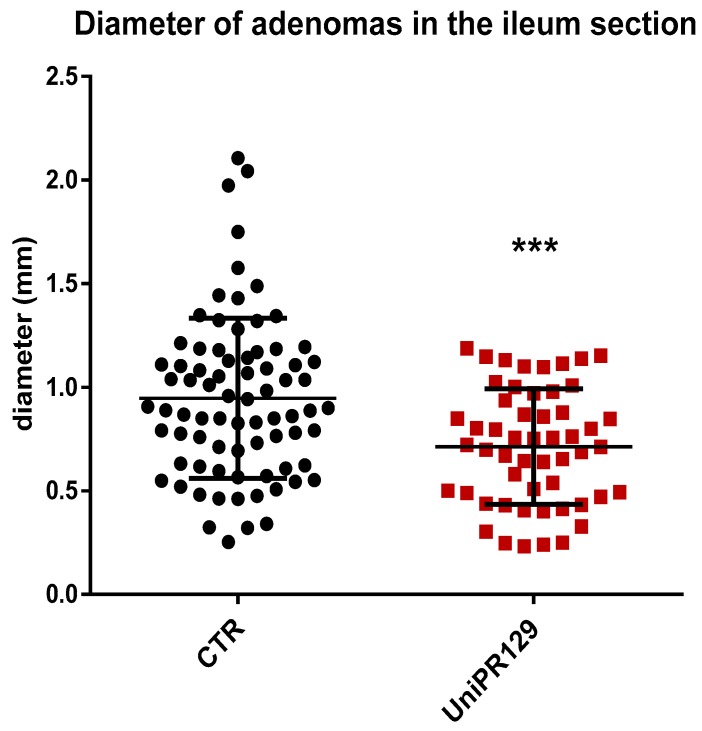
Diameter of all the adenomas collected in the Ileum of APC ^min^/J CTR mice and after oral administration of UniPR129 30 mg/kg. Mann–Whitney test was performed to compare tumor diameter of control to UniPR129 group (*** *p* < 0.001). Black dots: control; red squares: UniPR129.

Moreover, analyzing the diameter of each single adenoma raised in the ileum, it emerged as mice of UniPR129 treated group possessed smaller adenomas than the ones of the control group, (Figure 4). Indeed, the mean adenoma diameter was 0.94 mm ± 0.39 (SD) for the control group and 0.71 mm ± 0.28 (SD) for UniPR129 treated mice. 

**Figure 5:**
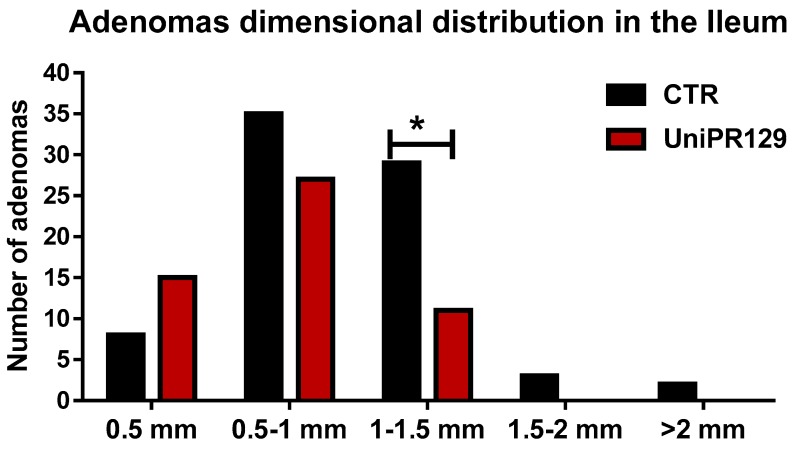
Adenomas dimensional distribution for control and UniPR129 group. Two-way ANOVA followed by Bonferroni’s post-test was used to compare control group to UniPR129 group. * *p* < 0.05, n = 7.

After that, all the adenomas grown in the ileum were pooled in classes, based on the diameter. Thus, 5 classes were identified (1 very small < 0.5 mm, 2 small from 0.5 to 1 mm, 3 medium from 1 to 1.5 mm, 4 large from 1.5 to 2 mm, 5 very large > 2 mm) and the number of adenomas was counted in each group. A different dimensional distribution was observed comparing the control group with mice treated with UniPR129 (Figure 5). In fact, the number of medium adenomas was significantly lower in UniPR129 group (11 ± 1.3 (SD) vs. 29 ± 2.5 (SD) of CTR group) and only in control mice compared some large (3 ± 0.5 (SD)) and very large adenomas (2 ± 0.5 (SD)). Conversely in UniPR129 group besides not having large and very large adenomas, most adenomas were categorized like small (51%) and very small (28%) (Table 4). Overall these data suggest a beneficial effect of UniPR129 treatment against the progression of the disease.

## 3. Discussion

The role of Eph-ephrin system in colorectal cancer has been investigated by several researches. EphA1 was found upregulated in locally invasive CRC but down-regulated in metastatic CRC and thus proposed as a prognostic marker [9]. EphA2 overexpression has been observed in tumor intestine compared to normal intestine, together with a change in the spatial expression of this receptor [7,15]. In fact, EphA2 is physiologically expressed in differentiated non-proliferative cells in the small intestine and in intercrypt tables of the large intestine, while it becomes constantly and evenly expressed over the whole intestine in case of tumors, in proliferative cells too [15]. As small intestine and colon tumors are believed to arise from intestinal stem cells, hence the hypothesis that EphA2 could be involved in tumorigenesis. Dunne et al. [8] observed a positive correlation between EphA2 and the stem cell markers CD44 and Lgr5, in tumor samples deriving from patients with colorectal cancer. 

Analysis of data was a little bit cumbersome since, as explained in statistics section, the different experimental groups showed dissimilar variances and different data distributions. For this reason, we split the analysis of any single group and compared it to control. First of all, the large variance of data obtained in the UniPR139 group hampers any reliable comment on the efficacy of this compound whereas UniPR1331 appears to be inactive probably because it undergoes extensive metabolism by gut microbiota [24]. UniPR129 was instead the only compound to show positive results in our model of intestinal tumorigenesis. In fact, the administration of the Ephs antagonist UniPR129 in APC ^min^/J mice reduced of the 30% the number of adenomas in the ileum, as well as, it decreased the diameter of adenomas in the same region. These results further confirm literature data about the oncogenic role of EphA receptors in intestinal cancer. Notably, consistent with our observations, it was previously demonstrated that the loss of EphA2 in APC ^min^/J mice led to a reduction of 33% of the tumor multiplicity in the small intestine and of the 60% and in the large intestine [15]. Thus, the block of EphAs signaling might be useful to delay the tumor development. 

We should also consider that beside the tumor-promoting role of EphA receptors, the EphB-ephrin-B system would possess a tumor suppressor role in colorectal cancer [25]. In fact, APC ^min^ mice with a single allele inactivation of EphB4 showed shorter lifespan, larger tumor size in the small intestine and more abundant tumors in the large intestine [26]. Moreover, it was observed as the spreading of intestinal EphB2-EphB3-positive tumor cells was hampered by the interaction with surrounding ephrin-B1-expressing normal cells, which led to the reinforcement of E-cadherin adhesion [25]. As UniPR129 is a pan Eph-ephrin inhibitor [16], it might also interfere with EphB-ephrin-B signaling and thus potentially contribute to tumor growth. However, our positive data may suggest that in APC ^min^/J mice adenomas development the EphAs signaling could have a more prominent role than EphBs signaling at least in the earliest phase of the tumor development.

As we previously said, APC ^min^/J mouse model is not totally representative of human colorectal cancer and it possesses notable differences, as the presence of small intestinal lesions, contrary to the majority of tumors being in the colon and rectum of familial adenomatous polyposis (FAP) patients. For these reasons, it could be interesting to test UniPR129 in a model of colorectal cancer especially if we consider the conflicting data from the literature. Indeed, EphB4 receptors have shown to own a tumor promoting role in advanced phases [10]. Nude mice injected with HT-29 cells, a line of human adenocarcinoma, showed reduced tumor growth and tumor angiogenesis after EphB4 suppression. Conversely there are clinical data showing that EphB3 and EphB2 expression is significantly reduced in advanced and spread colorectal tumors, suggesting a tumor suppressive function for these receptors [11,27,28]. Finally, other reports have shown the heterogeneous expression of Eph receptors not only between the different stages of CRC but also within the CRC specimens. EphA1, EphA2, EphB2 and EphA7 appeared as the receptors with the greatest variability of expression [9,27,29]. As suggested by Herath et al. the identification of which Eph are highly or least expressed should be performed on individual CRCs, in order to define which could be potential targets. 

These results, according to literature, further suggest Eph kinases as a new valuable target for delaying FAP progression. However, additional studies with new pharmacological tools interfering with Eph-ephrin system will be useful to dissect the contribution of the different Eph receptor (or ephrin ligand) subtypes and to understand the role played by both the forward and the reverse signaling in the intestinal tumorigenesis.

## 4. Materials and Methods 

Animal experiments received the approval of local Animal Care Committee and Italian Ministry of Health (approval code 212/2015-PR) and mice were used in compliance with European Community Council Directive 2010/63/UE and Italian regulation (DL 26/2014).

### 4.1. APC ^min^/J Mice

5 weeks old C57BL/6J-APC ^min^/J male mice were purchased from Jackson Laboratory (Bar Harbor, ME USA). Mice were weighted and randomly subdivided in four groups:

- Control group: Oral administration of Methocel 0.5% every other day;
- Three treatment groups: oral administration of UniPR129, UniPR139 or UniPR1331 30 mg/kg in Methocel 0.5% every other day.

The experiment was carried out for 8 weeks and over this period the weight was monitored once a week. At the end of the experiment, all mice were sacrificed by carbon dioxide inhalation and blood samples were collected via intracardiac injection and used for evaluation of hematological parameters. 

### 4.2. Histological Analysis

The whole intestine of all the mice was resected, washed with sterile PBS and fixed with 4% formalin. After 24 h, the intestine was longitudinally divided in 6 portions (1-duodenum, 2-jejunum, 3-ileum, 4-caecum, 5-colon, 6-rectum) and each portion was embedded in paraffin and sectioned at 5 μm with microtome (Leica). Slides were dried at 37 °C for 24 h, then deparaffinized in xylene, rehydrated with decreasing concentration of alcohol and stained with hematoxylin and eosin.

Histological slides were examined with Nikon Eclipse E800 microscope (Nikon Corporation, Japan) and photographs of sections were taken at 2x with Camera Nikon DIGITAL SIGHT attached to the microscope. The observation of longitudinal sections of the small intestine allowed to determine the distribution and number of adenomatous lesions; four longitudinal sections have been prepared for each segment of small intestine. The microscopic images of the ileum have been subsequently analyzed, using the ImageJ software, to determine the diameter and area of each adenoma.

### 4.3. Statistical Analysis

Hematological and blood chemistry parameters were undergone to one-way ANOVA to compare control group to treatment groups. Details on statistical analysis are reported in the “results” Section 2.2 “statistical consideration”. * *p* < 0.05, ** *p* < 0.01, *** *p* < 0.001.

## Figures and Tables

**Table 1:** Haematological parameters and blood chemistry. One-Way ANOVA was used to compare the control to all other groups for each parameter. SD = standard deviation. * *p* < 0.05, n = 7.

		CTR	UniPR129	UniPR139	UniPR1331
		Mean ± SD	Mean ± SD	Mean ± SD	Mean ± SD
White cells	103/μL	5.53 ± 1.67	4.83 ± 0.70	4.67 ± 1.19	4.70 ± 1.33
Neutrophils	%	2.64 ± 1.54	6.20 ± 6.33	12.36 ± 12.2 *	3.40 ± 0.87
Lymphocytes	%	97.10 ± 1.56	93.29 ± 6.14	87.33 ± 12.16 *	96.23 ± 0.83
Monocytes	%	0.17 ± 0.08	0.43 ± 0.31	0.11 ± 0.11	0.3 ± 0.20
Eosinophils	%	0.07 ± 0.05	0.10 ± 0.10	0.09 ± 0.09	0.07 ± 0.05
Basophils	%	0.01 ± 0.04	0.00 ± 0.00	0.00 ± 0.00	0.00 ± 0.00
Red cells	106/µL	5.62 ± 1.05	5.01 ± 1.43	5.99 ± 1.45	5.22 ± 1.14
Haemoglobin	g/dL	9.70 ± 1.49	8.71 ± 2.15	9.99 ± 1.79	9.20 ± 1.53
Haematocrit	%	29.59 ± 4.06	26.69 ± 6.56	30.11 ± 4.44	27.83 ± 4.25
Mean cellular Volume	fL	52.99 ± 2.85	53.87 ± 2.62	51.57 ± 6.6	54.1 ± 4.99
Platelets	103/µL	1148 ± 244.71	1191 ± 186.77	993.29 ± 344.17	1224.29 ± 220.88
Mean platelet Volume	fL	5.01 ± 0,42	4.91 ± 0.43	5 ± 0.21	4.97 ± 0.39
Creatinine	mg/dL	0.25 ± 0.07	0.22 ± 0.08	0.19 ± 0.02	0.24 ± 0.05
AST/GOT	UI	118.14 ± 55.82	108.86 ± 47.11	91.29 ± 20.70	100.71 ± 22.90
ALT	UI	30 ± 11.87	23.43 ± 3.78	22.29 ± 4.54	22.71 ± 5.71
Glucose	mg/dL	155.22 ± 27.98	161.10 ± 21.18	122.05 ± 27.52	132.92 ± 21.95
Triglycerides	mg/dL	156.3 ± 51.70	145.10 ± 38.6	200.61 ± 173.26	133.27 ± 88.98

**Table 2:** Summary statistics of control and treated groups. SD = standard deviation.

		Overall Intestine Adenomas		Ileum Adenomas	
	Obs	Mean	SD	Mean	SD
CTR	7	19.71	6.10	13.14	5.05
UniPR129	7	14.00	2.31	7.57	2.07
UniPR139	7	15.57	15.22	10.14	9.41
UniPR1331	7	18.71	6.15	9.86	5.49

**Table 3:** *p*-values of two-tailed *t*-tests for pairwise comparisons of the mean number of adenomas.

	Overall Intestine		Ileum	
	Equal Variance	Unequal Variances	Equal Variance	Unequal Variances
CTR—UniPR129	0.039	0.050	0.019	0.027
CTR—UniPR139	0.517	0.523	0.472	0.476
CTR—UniPR1331	0.765	0.765	0.266	0.267

**Table 4:** Percentage of adenomas for each class calculated on the total number of adenomas in the ileum section.

Treatment	Very Small	Small	Medium	Large	Very Large
CTR	10%	45%	38%	4%	3%
UniPR129	28%	51%	21%	0%	0%
